# Microbiomes of the dust particles collected from the International Space Station and Spacecraft Assembly Facilities

**DOI:** 10.1186/s40168-015-0116-3

**Published:** 2015-10-27

**Authors:** Aleksandra Checinska, Alexander J. Probst, Parag Vaishampayan, James R. White, Deepika Kumar, Victor G. Stepanov, George E. Fox, Henrik R. Nilsson, Duane L. Pierson, Jay Perry, Kasthuri Venkateswaran

**Affiliations:** Jet Propulsion Laboratory, Biotechnology and Planetary Protection Group, California Institute of Technology, M/S 89-2 4800 Oak Grove Dr., Pasadena, CA 91109 USA; Department of Earth and Planetary Sciences, University of California, Berkeley, CA USA; Resphera Biosciences, Baltimore, MD USA; Department of Biology and Biochemistry, University of Houston, Houston, TX USA; Department of Biological and Environmental Sciences, University of Gothenburg, Gothenburg, Sweden; Johnson Space Center, Houston, TX USA; Marshall Space Flight Center, Huntsville, AL USA

**Keywords:** International Space Station, Air, Surface, Microbiome, Closed habitat, Cleanroom, Propidium monoazide

## Abstract

**Background:**

The International Space Station (ISS) is a unique built environment due to the effects of microgravity, space radiation, elevated carbon dioxide levels, and especially continuous human habitation. Understanding the composition of the ISS microbial community will facilitate further development of safety and maintenance practices. The primary goal of this study was to characterize the viable microbiome of the ISS-built environment. A second objective was to determine if the built environments of Earth-based cleanrooms associated with space exploration are an appropriate model of the ISS environment.

**Results:**

Samples collected from the ISS and two cleanrooms at the Jet Propulsion Laboratory (JPL, Pasadena, CA) were analyzed by traditional cultivation, adenosine triphosphate (ATP), and propidium monoazide–quantitative polymerase chain reaction (PMA-qPCR) assays to estimate viable microbial populations. The 16S rRNA gene Illumina iTag sequencing was used to elucidate microbial diversity and explore differences between ISS and cleanroom microbiomes. Statistical analyses showed that members of the phyla Actinobacteria, Firmicutes, and Proteobacteria were dominant in the samples examined but varied in abundance. Actinobacteria were predominant in the ISS samples whereas Proteobacteria, least abundant in the ISS, dominated in the cleanroom samples. The viable bacterial populations seen by PMA treatment were greatly decreased. However, the treatment did not appear to have an effect on the bacterial composition (diversity) associated with each sampling site.

**Conclusions:**

The results of this study provide strong evidence that specific human skin-associated microorganisms make a substantial contribution to the ISS microbiome, which is not the case in Earth-based cleanrooms. For example, *Corynebacterium* and *Propionibacterium* (Actinobacteria) but not *Staphylococcus* (Firmicutes) species are dominant on the ISS in terms of viable and total bacterial community composition. The results obtained will facilitate future studies to determine how stable the ISS environment is over time. The present results also demonstrate the value of measuring viable cell diversity and population size at any sampling site. This information can be used to identify sites that can be targeted for more stringent cleaning. Finally, the results will allow comparisons with other built sites and facilitate future improvements on the ISS that will ensure astronaut health.

**Electronic supplementary material:**

The online version of this article (doi:10.1186/s40168-015-0116-3) contains supplementary material, which is available to authorized users.

## Background

The microbial characterization of the International Space Station (ISS) has been mostly limited to traditional culture-based microbiology and selective molecular biology methods, such as Sanger sequencing, for supporting tasks such as water remediation, food safety, and crewmember health [1–5]. Since built environments are known to have specific microbiomes [6], it is of the highest interest to the National Aeronautics and Space Administration (NASA) scientific community to explore the environmental microbiome of the ISS as a closed environment. Moreover, the National Research Council (NRC) specifically recommended that NASA study changes in microbial populations in response to selective pressure associated with microgravity, which characterizes life aboard the ISS [7]. Previous studies show that permanent changes have occurred within the microbial species during experiments aboard the ISS [8, 9]. Although next-generation sequencing (NGS) analyses are now broadly implemented in many microbiology-related scientific fields, especially in microbial ecology [10, 11] and human microbiome projects [12, 13], use of these techniques for closed habitats has just begun [14] and warrants more research.

“Deep” sequencing of ISS samples can answer questions on abundance and diversity of the microorganisms. However, differentiating viable and yet-to-be-cultivable microbial populations requires an appropriate sample processing technology [15]. The use of the reagent propidium monoazide (PMA) before DNA extraction eliminates cells with a compromised membrane. The PMA-based assay thus allows for a more accurate approximation of the viable microbial community in terms of richness as well as abundance [15]. Due to the technically rigorous methods required for culturing many microorganisms, characterization of human-associated microbial populations in the ISS environment remains a significant challenge. However, it is important to monitor the presence of any opportunistic pathogenic microorganisms. As long-duration human missions are planned in the future, detection of human pathogens and possible mitigation practices must be developed. In addition, understanding of the ISS microbiome could facilitate the necessary maintenance of this closed habitat and thereby assist in preventing degradation of its components by some microorganisms [4].

Both ISS environment and cleanrooms on Earth are maintained by high-efficiency particulate arrestance (HEPA) filters. Importantly, considerable prior knowledge of the microbial communities associated with these cleanrooms exists from prior studies. The ISS has been exposed to these communities indirectly by the fact that some of the cargos sent to the ISS were packaged in these specific NASA cleanrooms. As in the case of the ISS closed habitat, the Earth-based cleanrooms are controlled for humidity, temperature, and circulation. Furthermore, NASA quality assurance engineers perform periodic audits to ensure that certified-facility cleanliness levels conform to the requirements delineated for both the ISS and Earth cleanrooms. The ISS environment is zero gravity with exposure to space radiation and elevated carbon dioxide levels. By necessity the air is re-circulated whereas on Earth the cleanrooms are constantly replenished with fresh air. An important difference is human habitation, which does not occur in the Earth cleanrooms. However, the human traffic in Earth cleanrooms is actually rather high (at least 50+ people in a given working day) when compared to the ISS where only 6 astronauts are allowed at a single time.

This study is the first to analyze samples from the ISS air and surface using the traditional (colony counts [i.e., cultivable bacteria]) and state-of-the-art molecular techniques (adenosine triphosphate [ATP] and quantitative polymerase chain reaction [qPCR] assays) to measure the abundance of microorganisms (i.e., live and dead cells). Furthermore, the abundance and diversity of viable bacterial community were assessed using molecular methods (PMA treatment followed by qPCR [i.e., bacterial burden] or Illumina-based 16S rRNA sequencing [i.e., microbial diversity]). Additionally, the microbial diversity of the ISS was compared with samples from Jet Propulsion Laboratory (JPL) spacecraft assembly facility (SAF) cleanrooms, which also represent closed and environmentally controlled built ecosystems.

## Results

### Microbial burden

The samples analyzed during this study were the following: ISS HEPA filter particulates, vacuum cleaner bag components of ISS (ISS Debris), JPL Class 10 K cleanroom (JPL-SAF Debris), and JPL Class 1 K cleanroom (JPL-103 Debris) (Table 1). Microbial burdens estimated via traditional cultivation-dependent and cultivation-independent methods are given in Table 2. The bacteria capable of growth in nutrient-rich media were in the range of 10^5^ CFU per gram in the ISS HEPA filter sample and an order of magnitude greater (10^6^ CFU per gram) for the ISS Debris. Relatively, the bacterial populations were larger in the ISS Debris sample than in the JPL cleanroom debris samples. When compared to the ISS Debris, the JPL-SAF Debris harbored an order of magnitude smaller bacterial population (10^5^ CFU per gram) and the JPL-103 Debris sample was two orders of magnitude fewer (1.4 × 10^4^ CFU per gram). In general, cultivable fungi were less represented than bacteria in all samples tested. The cultivable fungal population was two logs less for the ISS samples when compared to their bacterial counts, whereas the JPL cleanroom samples exhibited one-log difference. The ISS Debris possessed a similar fungal burden as that of the JPL-SAF Debris sample, which is higher than the more stringent JPL-103 area. The ISS HEPA harbored less fungi when compared to the ISS Debris.

**Table 1:** Characteristics of samples collected from ISS cabin locations and Earth-based cleanrooms where spacecraft components are assembled

Sample name	Replicates	Location	Cleanroom classification	Source	Air/surface	Specifications	Usage time	Model	Mission activities
ISS HEPA	1	ISS	None	Filter element particles	Air	HEPA rated, retains 99.97 % particles 0.3 μm; 20-mesh inlet screen has 841 μm sieve openings	40 months	Part no. SV810010-1, Serial no. 0049; HEPA media supplied by Flanders Filters, Inc.; Nomex inlet screen	Returned aboard STS-134/ULF6 in May 2011
ISS Debris	2	ISS	None	vacuum cleaner bag dust	Surface	Vacuum bag retains particles >6 μm; HEPA-rated filter retains particles >0.3 μm	1 day	ISS vacuum cleaner	Expedition 31; returned aboard Soyuz flight 29S in July 2012
JPL-SAF Debris	2	JPL-SAF	10 K	Vacuum bag dust	Surface	Retains 99.7 % particles >3 μm	70 days	Nilfisk GM80, 81620000	No major mission
JPL-103 Debris	1	JPL-103	1 K	Vacuum bag dust	Surface	Retains 99.7 % particles >3 μm	>180 days	Nilfisk GM80, 81620000	No major mission

**Table 2:** Total and viable microbiological characteristics of particles accumulated in ISS and other Earth-based cleanrooms

Sample	Cutivable bacterial population (CFU/g) that are:		qPCR-based bacterial population (16S rRNA copies/g)		Viable bacterial population (B/A ×100)	ATP-based microbial population (RLU/g):		Viable microbial population (D/C ×100)
	Bacteria	Fungi	Untreated (A)	PMA-treated (B)		Total ATP (C)	Intracellular ATP (D)	
ISS HEPA	8.17 × 10^5^	2.34 × 10^3^	1.7 × 10^9^	2.91 × 10^7^	1.7	2.06 × 10^7^	3.54 × 10^5^	1.7
ISS Debris	1.34 × 10^6^	5.02 × 10^4^	4.5 × 10^8^	1.21 × 10^7^	2.7	3.50 × 10^7^	2.65 × 10^7^	75.7
JPL-SAF Debris	1.28 × 10^5^	5.05 × 10^4^	3.1 × 10^8^	2.07 × 10^8^	66.8	1.91 × 10^7^	2.10 × 10^6^	11.0
JPL-103 Debris	1.40 × 10^4^	3.30 × 10^3^	4.3 × 10^8^	1.90 × 10^7^	4.5	2.60 × 10^6^	1.20 × 10^6^	46.2

The qPCR-based assay, which measured DNA from both dead and live cells, estimated that the ISS HEPA sample had the highest bacterial density (1.7 × 10^9^ 16S rRNA copies per grams) compared to all other samples, which were at least one-log less abundant in total bacterial burden. This trend was not noticed in the samples that were treated with PMA (viable bacterial population), where ~10^7^ 16S rRNA gene copies per gram accounted for all samples except in the JPL-SAF, which exhibited ~10^8^ 16S rRNA gene copies. The percentage of the viable bacterial population (PMA-treated samples) was ~1.7 % in the ISS HEPA, ~2.7 % in ISS Debris, and ~4.5 % in the JPL-103 samples. However, approximately 67 % of the bacteria in the JPL-SAF Debris were viable.

The total ATP contents derived from both dead and alive cells were in the range of 10^7^ relative light unit (RLU) per gram, except for the sample collected from the Class 1 K JPL-103 cleanroom, which showed ~2.6 × 10^6^ RLU per gram. The microbial estimation carried out via an intracellular ATP assay (viable cells only) indicated that only 1.7 % of the total microorganisms were viable in the ISS HEPA sample. In the case of the ISS Debris sample, this percentage was much higher (75 %); similarly, JPL-SAF and JPL-103 constituted approximately 11 % and 46 % viable microorganisms, respectively.

### Culture-based microbial diversity

The 16S rRNA sequencing-based identification and phylogenetic affiliation of the bacterial and fungal strains isolated during this study are given in Additional file 1: Table S1. Among 41 bacterial strains isolated and identified, 36 belonged to 29 different species, five strains were only identified to the genus level. The majority of the identified isolates belong to Firmicutes and only four strains were affiliated to the members of the Proteobacteria group. The highest number of isolates was represented by *Bacillus* and *Staphylococcus* genera. While *Bacillus* was dominant in both ISS samples, *Staphylococcus* was only present in ISS Debris. Both genera were either absent or underrepresented in the JPL-SAF and JPL-103 samples. Single representatives of other spore-forming lineages were also identified (*Paenibacillus*, *Brevibacillus*, and *Solibacillus*). Notably, five strains belonging to the *B. anthracis-cereus-thuringiensis* group were isolated from the HEPA filter, and more detailed phylogenetic, pathogenic, and whole genome sequence characterizations are underway to confirm the functional characteristics of these isolates. The pathogenic properties specific to *B. anthracis* were not present in these five strains (data not shown).

Among 19 fungal strains sequenced, 13 strains were identified to six different species. Out of 19 fungal strains, 16 were isolated from the ISS samples and the other three strains were from JPL samples. All the cultivated fungal strains affiliated phylogenetically to the members of the *Ascomycota* phylum. The most common fungal isolate in the ISS samples was *Aspergillus niger*, although other diverse species of *Aspergillus* were also identified, along with *Penicillium* as the second most dominant genus (Additional file 1: Table S1). Based on these results, most of the cultivated bacteria or fungi from the ISS locations were not noticed in the Earth-analog cleanrooms.

### Pyrosequencing-derived bacterial diversity

The number of pyrosequences belonging to various bacterial phyla and operational taxonomic units (OTUs) is presented in Additional file 1: Table S2. Approximately, 100,000 reads of bacterial pyrosequences of >500 bp in length were generated from eight samples during this study. When the software mothur [16] was employed for the bioinformatics analysis, ~71 % of the sequences were deemed to be of good quality and used for further analysis, resulting in 70,669 sequences (Additional file 1: Table S2).

Among PMA-untreated samples, the ISS Debris yielded the highest number of sequences (30,111 reads) whereas the lowest number of sequences was seen in the ISS HEPA (1,720). Likewise, more sequences were retrieved from the JPL-SAF (8,482 reads) as compared with the JPL-103 (5,062) sample. Contrary to the pyrosequence abundance, higher numbers of OTUs were present in the JPL-SAF sample (1,448 OTUs) than in the ISS Debris (452 OTUs) sample. However, as noticed in the pyrosequence abundance, the number of OTUs was also higher in the JPL-103 sample (301 OTUs) as compared to the ISS HEPA (133 OTUs) sample.

The PMA-untreated samples constituted ~65 % of the high-quality sequences, while only 35 % of the sequences were retrieved from PMA-treated samples (25,294 sequences). Among the PMA-treated (viable) portions, ISS HEPA and ISS Debris samples possessed ~38 % and 60 % of sequences, respectively. Similar to the ISS Debris sample, a higher percent of viable sequences was retrieved from the JPL-SAF Debris (77 %) but surprisingly, only ~3 % of the viable sequences were from the PMA-treated JPL-103 Debris sample.

Almost all of the viable bacterial pyrosequences retrieved from the ISS HEPA filter were members of *Corynebacterium*. This organism was also dominant in the ISS Debris sample (Additional file 1: Table S2). Genera such as *Propionibacterium* and *Staphylococcus* were only present in the ISS Debris sample. While the ISS-HEPA sample contained fewer sequence reads representing only five genera, the ISS Debris sample was dominated by low abundance of more diverse genera (*Stenotrophomonas*, *Sphingomonas*, *Pseudomonas*, *Delftia). Bacillus* species were predominant when traditional cultivation methods were employed (Additional file 1: Table S1), but *Bacillus* pyrosequences were not retrieved. When PMA-treated samples from the ISS samples were compared to the Earth-analog cleanroom samples, the dominant phyla were found to be very similar (Actinobacteria, Proteobacteria, Firmicutes). However, on the genus level, the JPL-SAF yielded many more viable sequences representing diverse genera (data not shown).

### Illumina-derived bacterial diversity

**Fig. 1:**
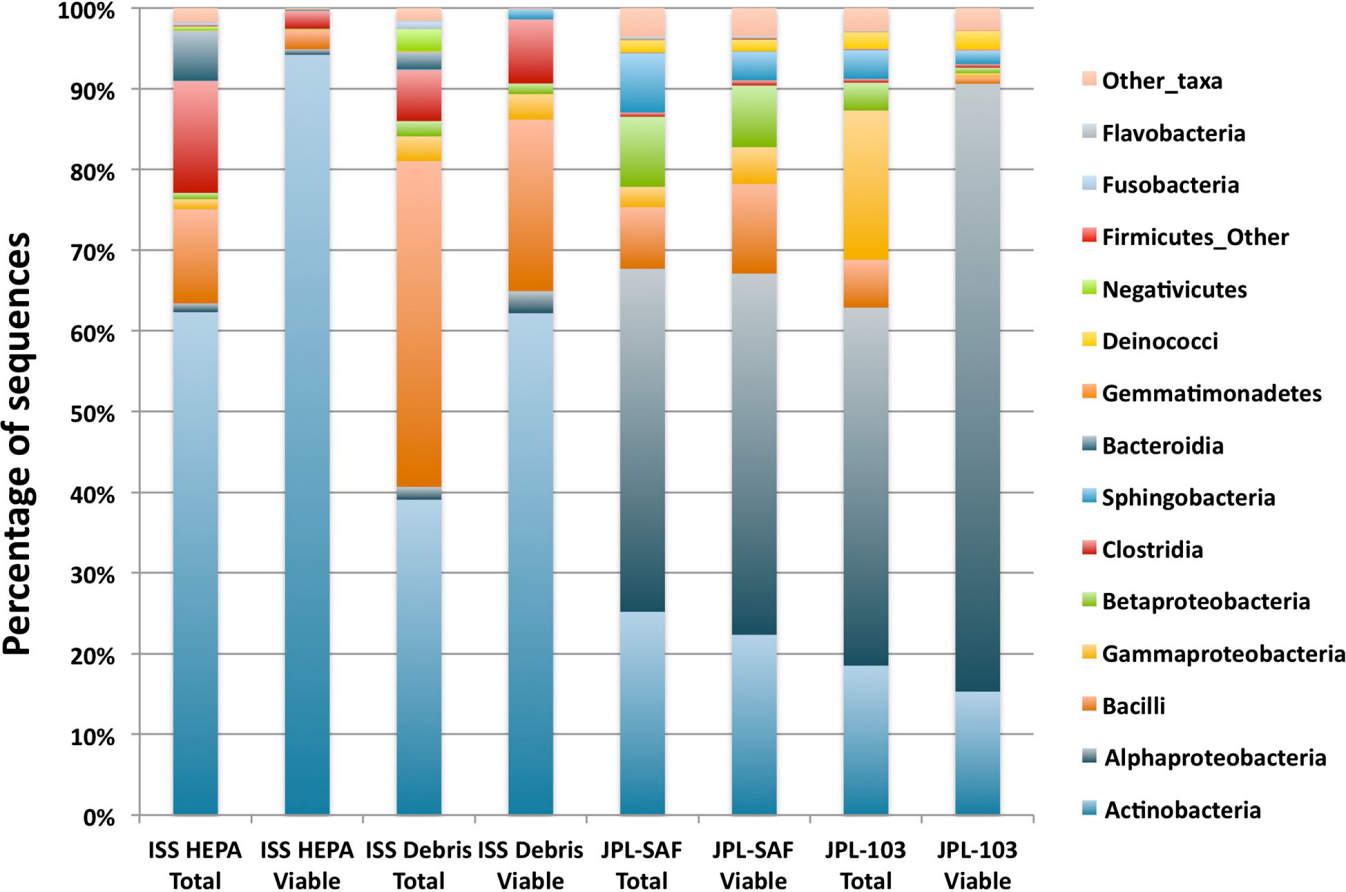
Taxonomic profiles of R1 samples at the class level

The Illumina sequencing generated ~6.8 million high-quality reads, which is ~100 times more coverage than was obtained with pyrosequencing yield Additional file 2: Figure S1. Since Illumina sequences were short (~150 bp each), it was not possible to reliably resolve taxonomic affiliation beyond the genus level. We characterized high-quality reads using the Ribosomal Database Project (RDP) classifier [17] and summarized community composition at multiple taxonomic levels. Among the ISS-associated samples, profiles were dominated by Actinobacteria, Bacilli, and Clostridia, while samples from the JPL-associated sites maintained higher levels of Alphaproteobacteria and Gammaproteobacteria (Fig. 1). At the family level, the differences between ISS and JPL profiles were substantial, with complete distinction between the two sites using actinobacterial members alone—the ISS samples were dominated by *Corynebacterium*, while the actinobacterial groups among JPL samples were predominantly *Geodermatophilaceae*.

Actinobacteria, Firmicutes, and Proteobacteria phyla together constituted 80 % or more in all cases. When compared on the family level, sequences associated with any family did not exceed more than 10 % of the total sequences. Characteristics of bacterial phyla that are dominant during this study are depicted in Table 3. A clear-cut trend was noticed on the presence of bacterial phylum dominance on the ISS as well as in the Earth-analog cleanroom samples. Viable sequences arising from Actinobacteria were the most abundant in the ISS HEPA and ISS Debris samples (~95 and ~66 %, respectively), while Proteobacteria was present in the least numbers (0.41 and ~3 %, respectively). Among these three dominant phyla, the lowest percentage was found for Firmicutes and differed significantly between cleanroom samples as well (JPL-SAF: 11.05 % and JPL-103: 0.98 %). In-depth analysis showed that the ISS HEPA sample accumulated more sequences of the genus *Corynebacterium* than ISS Debris sample. In addition to the latter, the ISS Debris had sequences associated with the order Actinomycetales and the genus *Staphylococcus*. In contrast, the JPL-SAF Debris sample possessed larger numbers of *Bacillus* and unclassified Proteobacteria genera sequences, whereas JPL-103 Debris mimicked ISS locations and members of Actinobacteria were more dominant.

**Table 3:** Characteristics of dominant bacterial phyla as measured by Illumina iTag method during this study

	ISS HEPA total	ISS HEPA viable	ISS Debris total	ISS Debris viable	JPL-SAF total	JPL-SAF viable	JPL-103 total	JPL-103 viable
Total number of sequences	553,176	587,569	1,148,047	1,116,419	1,029,984	1,472,777	489,047	397,607
Percentage of sequences that belong to three dominant phyla	90.92	99.65	92.35	98.26	80.25	81.84	87.96	90.46
Actinobacteria								
Percentage of sequences	63.28	95.28	40.52	66.54	28.76	25.25	23.79	21.46
Number of genera	78	55	62	38	122	116	103	71
Number of dominant genera (>100 sequences)	16	7	28	16	71	76	28	24
Firmicutes								
Percentage of sequences	24.83	3.97	45.67	28.48	7.70	11.05	6.60	0.98
Number of genera	118	67	100	31	152	150	101	53
Number of dominant genera (>100 sequences)	50	17	65	18	53	69	15	7
Proteobacteria								
Percentage of sequences	2.81	0.41	6.16	3.24	43.80	45.55	57.57	68.02
Number of genera	95	65	89	30	189	191	143	92
Number of dominant genera (>100 sequences)	22	7	49	10	96	104	56	29

**Fig. 2:**
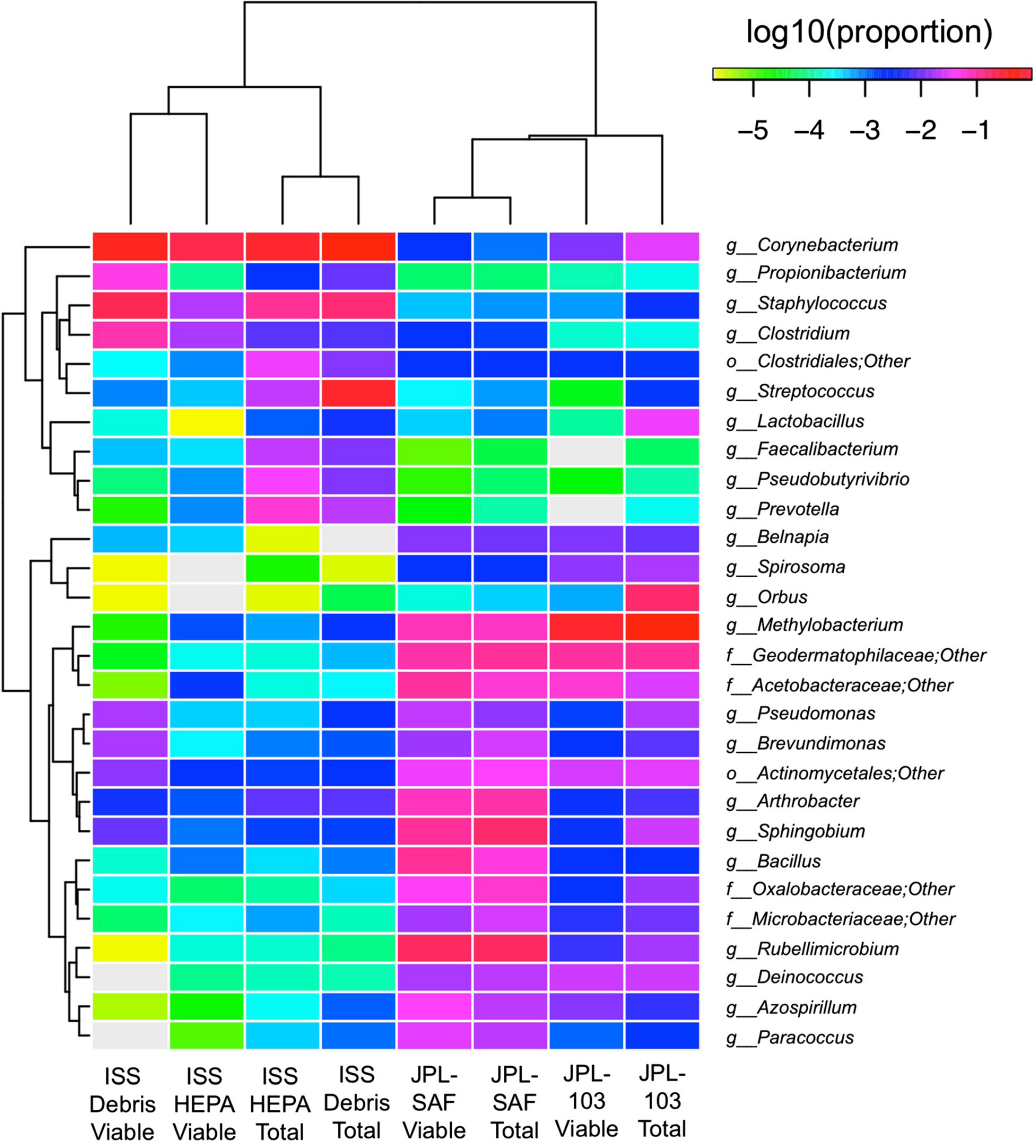
Hierarchical clustering of samples using taxonomic profiles at the genus level (R1 dataset). The taxonomic profiles clustered based on sampling location. The *color scale* reflects log-normalized proportional values (e.g. −1 ~ 10 %, −2 ~ 1 %, −3 ~ 0.1 %). JPL-SAF samples clustering together and showing several unique low abundance members were not found in the JPL-103 Debris and ISS samples (e.g., *Mollicutes*, *Nitrospira*, and members of *Chloroflexi*). *Rows* and *columns* are clustered independently using the furthest neighbor algorithm with a Euclidean distance metric. *o* order, *f* family, *g* genus

**Fig. 3:**
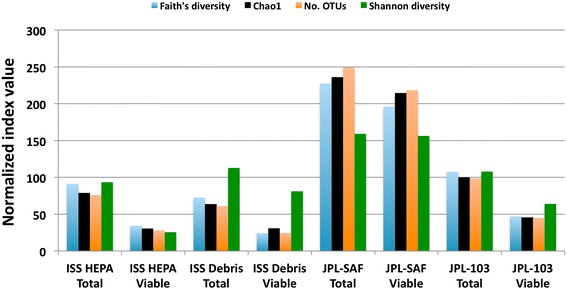
PMA treatment is associated with a reduction in alpha diversity. Alpha diversity values are normalized as a percentage of their mean value across all eight samples in the chart

In order to detect differentially abundant taxonomic groups between PMA-treated and untreated samples at the phylum through genus levels, several statistical analyses were conducted (see “Methods” section). Hierarchical clustering of samples using genus taxonomic profiles resulted in clustering of samples by collection site but not strong clustering of paired PMA or no PMA samples (Fig. 2). Both the negative binomial test and Fisher’s exact test provided valuable information and supported the hierarchical clustering scenario. Searching for differentially abundant genus-level groups with significant *P* values using the negative binomial test, we saw that the vast majority of differentially abundant taxa were relatively depleted in the PMA-treated group. The only well-represented genera that were relatively enriched were *Clostridium* (0.34 % vs. 2.46 %, *P* = 0.009) and *Rheinheimera* (0.005 % vs. 0.472 %, *P* = 7E-06). In contrast, genera such as *Streptococcus*, *Veillonella*, *Lactobacillus*, *Bifidobacterium*, and *Neisseria* were all relatively enriched in the untreated samples. Alpha diversity estimators also indicated a significant drop in diversity associated with PMA treatment. On average, PMA samples had 48 % fewer OTUs than their untreated pairs (paired *T*-test *P* = 0.004). This consistent reduction in the PMA samples was also true for Faith’s diversity, Chao1, and Shannon diversity measures (Fig. 3) and is consistent with the depletion of many taxa identified by differential abundance analysis.

**Fig. 4:**
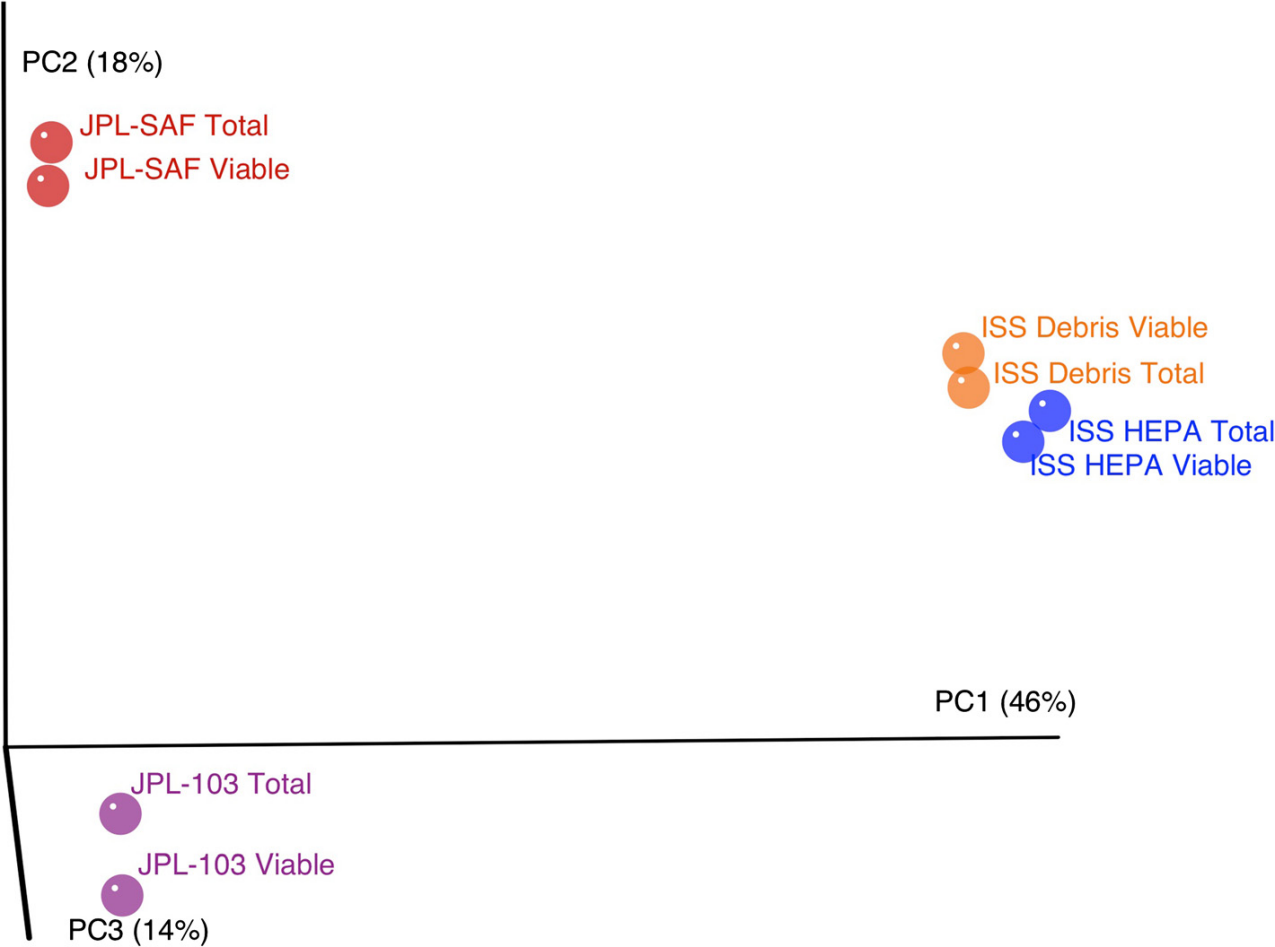
Principal coordinate analysis plot (R1 data) based on Bray-Curtis distances. Percentage of variance explained by each principal coordinate axis is shown in *parentheses*

To better characterize the shared community composition among samples, beta-diversity metrics (e.g., Bray-Curtis distances) were computed and evaluated using principal coordinate analysis (PCoA). Figure 4 displays PCoA plots associated with the R1 dataset. The samples from the same site are highly similar in composition regardless of PMA treatment. Using Procrustes analysis, we compared PCoA plots generated for both the R1 and R2 datasets. In this case, Procrustes analysis was employed to transform the R2-based principal coordinate set (by rotation, scaling, and translation) to minimize the distances between corresponding points in the R1 principal coordinate space. We found that virtually identical distances were derived between the R1 and R2 PCoA plots, further supporting the clustering of PMA and untreated paired samples. This high-resolution comparison stood in contrast to broader composition comparisons, such as the hierarchical clustering above. At the OTU level, the bacterial composition was strongly associated with the sampling site, and the reduced overall diversity of PMA treatment did not dramatically impact the composition relative to sample origin.

### Pyrosequencing-derived archaeal diversity

When the samples were subjected to the archaeal characterization, neither qPCR nor PCR for next-generation sequencing generated archaeal amplification products (data not shown). It is likely that the presence of archaea in these samples was at a very low concentration (<100 gene copies per PCR reaction), below the detection limits of the technology [14]. Alternatively, materials associated with the dust samples might have inhibited the archaeal DNA amplification. However, this is unlikely because the dust samples spiked with a purified archaeal DNA did exhibit an appropriate band after PCR amplification (data not shown).

### Pyrosequencing-derived fungal diversity

In total, ~35,000 fungal pyrosequences were retrieved from the eight samples. The detailed breakdown of the number of sequences and OTUs is given in Table 4. The comparison is made on the class level, as fungal taxonomy is not as well described on the genus level as bacterial taxonomy when it comes to sequencing of environmental substrates. The fungal OTUs represented 153 distinct taxa. Most OTUs were obtained for three classes: Dothideomycetes, Eurotiomycetes, and Tremellomycetes. As noticed for the bacterial abundance, fungal species richness was also location specific. The dry cleanroom surfaces were found to harbor different fungal species (mostly Dothideomycetes and Eurotiomycetes) whereas ISS samples possessed more Eurotiomycetes, Saccharomycetes, and Exobasidiomycetes members.

**Table 4:** Pyrosequencing-based fungal phyla present in ISS and Earth-based cleanroom samples

Phylum/class (number of genera)	Number of pyrosequences that are retrieved from:								
	Number of OTU	ISS HEPA total	ISS HEPA viable	ISS Debris total	ISS Debris viable	JPL-SAF Debris total	JPL-SAF Debris viable	JPL-103 Debris total	JPL-103 Debris viable
Ascomycota									
Dothideomycetes (25)	46	116		24	2681	2997	2089	1184	2362
Eurotiomycetes (7)	24	508		6278	1537	265	269	7	63
Leotiomycetes (2)	2					16			
Arthoniomycetes	8					51	29	1	
No rank	1					3			
Saccharomycetes (5)	6	2536		2859		46	5		
Sordariomycetes (11)	12			233		66	34		
Basidiomycota									
Agaricomycetes (3)	4			148		8			
Cystobasidiomycetes (3)	7					18	43	424	1020
Exobasidiomycetes	6	498		555				3	
Microbotryomycetes (3)	8	108		401		280	111	8	
Pucciniomycetes	3					2	18		
Tremellomycetes (5)	23			11	4110	204	175	90	186
Chytridiomycetes	2					4	1		
Early diverging fungal lineages	1					5	3		

When treated with PMA, no viable fungal sequences were retrieved from the ISS HEPA sample. In contrast, viable fungal sequences for Dothideomycetes and Tremellomycetes were common in the ISS Debris samples. Viable fungal diversity was far higher in the JPL-SAF Debris sample than in JPL-103 Debris. The JPL-SAF Debris sample revealed the highest diversity that are viable and the highest number of sequences. The great number of *Eurotiomycetes* sequences was confirmed by cultivation of *Aspergillus* and *Penicillium* isolates from the ISS HEPA and ISS debris.

### Significant differences of microbial communities between ISS and Earth cleanroom samples

The differences between the ISS and Earth-analog cleanroom microbiomes were assessed using multivariate statistics. Independent analyses of bacterial sequences derived from pyrosequencing and Illumina sequencing revealed a significant difference in the community profile of ISS and Earth-analog cleanroom microbiomes. These differences are displayed in ordination analyses (Additional file 3: Figure S2) and are supported by Permutational Multivariate Analysis of Variance (PERMANOVA) and Multi-Response Permutation Procedure (MRPP) analyses (Additional file 1: Table S4). Irrespective of the sample origin, the viable community profile was highly similar to the total community profile, revealing no significant differences in PERMANOVA or MRPP indices. However, the differences between the viable and total community profile were outweighed by the substantial differences of the ISS and Earth-analog cleanroom microbiome, which ranged from 0.1 to 0.5 concerning the chance-corrected within-group agreement (Additional file 1: Table S4). Bacterial and fungal taxa that showed a significant difference between ISS and Earth-analog cleanroom samples are presented in Additional file 3: Figure S2.

## Discussion

The safety and health of spaceflight crewmembers are of the highest importance for current and future missions. Individuals living and/or working in built environments are often susceptible to health issues associated with microorganisms [18, 19]. Moreover, the microbial ecology of ISS remains largely unknown, as study efforts have been mostly focused on microbiological surveillance using cultivation procedures. The NRC recommended use of state-of-the-art molecular biology techniques to develop better microbial monitoring of future closed habitat(s) and response system(s) against potential biohazards originating from microbiological sources, using the ISS as a test bed [1, 4]. Exploration of the microbial diversity associated with unusual built-in environments such as the ISS would further contribute to the indoor microbiome research. This will benefit the development of spaceflight applications as well as basic and applied research on Earth.

The main goal of this study was to determine ISS air and surface viable microbiomes and unveil the differences of viable microbiomes on the ISS and Earth-based cleanrooms. The ISS is a very unique environment due to microgravity, and a suitable Earth analog of this ecosystem is not available. The cleanrooms used for comparison in this study are environmentally controlled and represent oligotrophic conditions in an Earth-based setting. Comprehensive characterization and cataloguing of microbial species in NASA cleanrooms has been taking place since the 1970s. This extensive data set renders these facilities the best-characterized closed environments with limited human traffic that the ISS samples can be compared with [20–24]. A lower incidence of cultivable microorganisms in Earth cleanrooms than ISS surfaces suggests that regular thorough maintenance and cleaning practices are required to reduce microbial burden in closed habitats.

Multiple studies have used PMA for the estimation of viable bioburden, but they were mostly interpreted by qPCR [25–27], next-generation sequencing [15, 28, 29], or PhyloChipG3 [15]. The utilization of PMA combined with Illumina MiSeq technology for exploring viable microbiome of ISS-related environmental surfaces is not yet reported. The NGS technology resulting in information on millions of base pairs might have included genetic information from dead cells including contaminant DNA associated with the sample processing reagents [30, 31]. Thus, PMA treatment would eliminate these contaminants, making it possible to elucidate viable microbiomes. However, it was documented that PMA might not work for all kinds of microbial populations, especially spores where the dye was not able to penetrate the dead but intact spores [32]. Despite this, none of the other current methodologies can precisely estimate viable populations; PMA treatment has the highest potential to lead to the substantial differentiation between dead and live cells [15]. The statistical analysis of the viable bacterial community in this study revealed the significant variance in viable microbiome (Fig. 4; Additional file 1: Table S3 and S4), between the ISS and the cleanroom samples.

The ISS HEPA filter was in service for ~40 months whereas debris collected from the ISS surface was only 1 day old (Table 1). The HEPA filter system employed in ISS was designed to revitalize the air by passing through a heat-exchange process. Hence, particulates associated with ISS HEPA filter might have been exposed to drier conditions than the crew living quarter debris (relative humidity >50 %) and therefore the microorganisms that could withstand dry conditions only survived in the HEPA filter. Any microbial species that could withstand a prolonged period of time (~40 months during this study) requires survival capabilities against harsh conditions, and members of the Actinobacteria phylum are known to withstand desiccation, dry conditions, and high pH [33, 34], which explains their abundance in ISS HEPA filters (~95.28 %; Table 3). Comparatively, sequences of viable Actinobacteria were retrieved in more numbers in ISS surface debris (~66 %) than in Earth cleanroom debris (~25 %) samples.

The members of Actinobacteria phylum represent human skin commensals and also soil-borne microorganisms [35]. The dominance of Actinobacteria might be due to the presence of astronauts who live and perform work activities on board the ISS. However, characterization of astronauts’ skin microbiome is warranted and such studies will add more insights into the source of these bacteria associated with the ISS environmental surfaces and atmosphere. Actinobacteria have been previously reported in spacecraft assembly cleanrooms, and these non-spore-forming bacteria were considered a potential contamination risk of spacecraft due to their resistance to desiccation and ultraviolet radiation [34]. In this study, the viable population of the Actinobacteria did not constitute high population of the Earth cleanrooms, although this can be explained by the different gowning procedures required before entering these facilities unlike practiced in the ISS. In this study, both the ISS HEPA and ISS Debris samples contained high number of viable *Corynebacterium* sequences whereas *Propionibacterium* sequences were more in the ISS Debris sample. Since these microorganisms require special growth conditions, they were not isolated when traditional cultivation methods were employed during this study. Although the *Propionibacterium* species represent natural skin commensals, *P. acnes* is considered an opportunistic pathogen that leads to various infections [36, 37]. Similar concerns refer to *Corynebacterium*, which has received attention for being a genus containing several opportunistic pathogens [38]. Even though viable sequences of these opportunistic pathogens were retrieved from both ISS locations, their virulence characteristics are to be studied before correlating their influence on human health in a closed habitat. The risk of acquiring infection from opportunistic bacterial and fungal pathogens might pose a threat to crewmembers’ health and needs to be studied in the future.

In contrast, both of the Earth-based cleanrooms contained more Proteobacterial members when compared to the ISS locations. This trend has previously been seen in the studies of cleanroom-associated microbiomes when Sanger sequencing or DNA microarray method(s) were attempted, where human-associated Proteobacteria constituted the dominating phylum [11, 15, 39]. Low abundance (<100 sequences) of Gammaproteobacteria, human commensals, in ISS samples appears reasonable since at any given time only a maximum of six astronauts are present [40]. Despite more human traffic in Earth cleanrooms, lower abundance of Gammaproteobacterial members were noticed. This might be due to the fact that appropriate countermeasures (cleaning) were in place to prevent contamination in these indoor facilities. The high abundance of *Acinetobacter* and *Brevundimonas* in the ISS Debris sample showed that these microorganisms are ubiquitous in nature and associated with the debris [41] suggesting that their presence cannot be avoided. The members of the oligotrophic class *Sphingomonodaceae* were more abundant in the JPL-SAF sample and were previously found to persist in these nutrient-deprived environments [42].

Among the *Firmicutes*, species belonging to the genus *Staphylococcus* were common on the ISS surfaces but not present in the ISS air. This indicates that co-inhabiting astronauts might have shed more skin microorganisms that were captured by the vacuum cleaner than the HEPA system. The cultivation-dependent method confirmed the presence of several *Staphylococcus* species in the ISS surface samples and these species were not isolated from other samples investigated during this study. In contrast, the higher abundance of viable *Bacillus* species in the JPL-SAF (Class 10 K) sample suggests that they might have originated from the surrounding soil [43]. Lower abundance of *Bacillus* species in the more stringent JPL-103 cleanroom (Class 1 K) further reiterated that appropriate maintenance could regulate microbial presence in a much cleaner indoor environment [42].

The dominant cultivable isolates of bacteria from both ISS air and ISS surfaces were spore-forming *Bacillus*. As members of this genus are common inhabitants of soil and dust, they were highly probable to be cultured from the ISS samples on a nutrient-rich media. When the phylogenetic affiliation results for the cultivable strains were compared to the NGS results, only Illumina-derived deep sequencing yielded *Bacillus* sequences. In our previous efforts, *Bacillus* eluded detection when traditional Sanger sequencing [44], PhyloChip G2 technology [45], or 454-pyrosequencing analysis of amplicons were employed [11]. Likewise, in this study, the pyrosequencing method again did not retrieve sequences of spore formers. In contrast, the Illumina method frequently yielded the sequences indicative of the spore formers, including members of the genus *Bacillus* and *Paenibacillus*. The absence of *Solibacillus* and *Brevibacillus* sequences might be either attributed to the specificity of the iTag primer sets or the efficiency of the DNA extraction method employed. However, the Promega Maxwell-16 DNA extraction system used in this study was successful in extracting nucleic acids from the pure cultures of *Solibacillus* and *Brevibacillus*. Despite the same sample preparation, the Illumina-based sequencing generated more reads (40–2400 times; average ~100 times), thus providing a deeper coverage of microbial diversity than the 454 platform. Choosing an appropriate technology is important when abundance and richness of the microbial diversity are considered. Although Illumina can be employed to understand yet-to-be cultivated microorganisms, the use of well-established traditional techniques should not be completely abandoned.

Fungal strains have been detected on the ISS multiple times as actively growing molds on the environmental surfaces or responsible microorganisms deteriorating the components of ISS hardware [4]. The abundance of the fungal molds in indoor environments was reported to be associated with invasive infections and allergies [46, 47]. Therefore, a high number of fungal isolates from the ISS locations confirmed a need for continuous monitoring of the ISS for fungal populations. *Aspergillus niger* was the predominant isolate, and although it does not have the potential to cause disease at the same rate as other *Aspergillus* species (*A. fumigatus*, *A. flavus*), it was correlated with pulmonary and ear infections [48]. *Aspergillus* species are repeatedly isolated in indoor microbiomes [49]. The detection of multiple isolates of *A. niger* is consistent with its frequent presence in built environments including ISS surfaces [50]. However, its survival capability, proliferation, and molecular alteration in response to ISS microgravity conditions need to be studied further. On the other hand, *A. niger* is known for production of many beneficial substances. The potential for production of novel beneficial secondary metabolites under microgravity conditions needs to be explored for use in diverse biotechnological and pharmaceutical fields [51, 52].

## Conclusions

This study provided important insight into the ISS microbial diversity with a focus on significant differences between the closed habitats of the ISS and Earth-based cleanrooms. The NGS data, supported by traditional microbiology techniques and selective molecular bioburden estimation assays, provided a preliminary but detailed report on the ISS environmental microbiome. Future work will be able to build on these initial results by determining how stable that environment is over time. In contrast with the JPL cleanrooms, the most common organisms in the ISS environment were members of the Actinobacteria that are frequently associated with humans. As on the ISS, human traffic is common in the cleanrooms, but clearly the more stringent procedures possible on Earth are more effective in eradicating these organisms. The current results provide a baseline for evaluating the effectiveness of future ISS procedural changes. This study also allowed an evaluation of the utility of coupling PMA treatment with NGS sequencing for more accurate estimation of viable population. The bacterial diversity of each sample decreased after the PMA treatment but this was strongly associated with the sampling site. Since exposure to viable organisms is best minimized, it is important to know which areas are most problematic and hence should be avoided and/or targeted for more stringent cleaning conditions. It is clear from the results presented here that viable cell studies with PMA can provide this information. It was also found that the relative numbers of various types of viable cells in each sample closely resembled the distribution obtained when all (dead and viable) cells were considered. This is likely to be a usual result unless the population at a sampling site has been recently and dramatically changed such that the prior dominant organisms have been replaced in only the pool of viable cells.

## Methods

### Sample characteristics

Particulate materials were collected from the ISS HEPA filter element (ISS HEPA) and vacuum cleaner bag components (ISS Debris) used aboard the ISS as well as at two cleanrooms at JPL (one each originating from the SAF and building 103, Pasadena, CA), during this study. Table 1 lists sample characteristics, usage time of the material collection devices or system(s), model, make, and cleanroom conditions where the devices were used. The materials collected using the HEPA filter system represented the air and the vacuum cleaner bag represented the surface locations.

### ISS HEPA

The environmental control system (ECS) aboard the ISS includes a distributed ventilation system that contains HEPA filter elements to remove suspended particulate matter from the cabin atmosphere and protect humidity control and air purification equipment from debris accumulation and biofouling [53]. Particle counts are not routinely measured aboard crewed spacecraft. A study conducted during space shuttle mission STS-32 (January 1990) found that the particle size distribution approximated a class 100,000 cleanroom for particles <2.5 μm, a class 400,000 cleanroom for particles 2.5 μm to <10 μm, and a class 3000 cleanroom for particles >10 μm [53, 54]. The particle size distributions are defined using cleanroom standard definitions (FED-STD-209 1992). The HEPA filters used aboard the ISS retain 99.97 % particles of 0.3 μm via a pleated borosilicate media. A 20-mesh (841-μm sieve openings) inlet screen located at the filter element’s face removes larger debris and fibers. The pleated, non-woven borosilicate HEPA filter media was installed by Flanders Filters, Inc. (Washington, NC) in the filter element housing. Flanders Filters, Inc.’s commercial “NaturalAire” cut-to-fit filtration media has the greatest similarity to the ISS HEPA filter media (http://flandersfilters.com/products/naturalaire/). Twenty-one filter elements are distributed throughout the ISS in several modules and astronauts replace them on a scheduled maintenance cycle ranging from 2.5 to 5 years, depending on the location. The part number of the HEPA filter system analyzed during this study was SV810010-1 and the serial number was 0049. The HEPA filter element analyzed during this study was manufactured in September 1998, installed in ISS on January 2008, and returned aboard space shuttle flight STS-134/ULF6 in late May 2011. This filter was installed in ISS Node 2 and was in service for 40 months, although the typical service time is only 30 months for this location. Typically, ground testing is performed to characterize a filter element’s pressure drop at various process air flow rates [53] after retrieval from the ISS, but in this case, the HEPA filter remained untouched in its shipment packaging from the time it was removed from the ISS until particulates were recovered at JPL for microbial characterization. The particulate materials collected from this sample were designated as ISS HEPA during this study.

### ISS debris

The vacuum cleaner bag contained debris that had collected on the HEPA filter inlet screens. The vacuum cleaner bag components are representative of the collected debris (lint, food particles, skin/hair, and miscellaneous debris) that had been airborne in the cabin but had collected over time on the filter element inlet screen. The molecular microbial community analysis for the ISS vacuum bag debris (ISS Debris) was previously documented [14]. These results were utilized here for comparative purposes. The previous molecular characterizations of the ISS Debris sample were extended here with Illumina-based deep sequencing using archived DNA aliquots [14]. Sample characteristics of the ISS Debris are described in Table 1. Briefly, during the period of ISS Expeditions 30 and 31, reports by some crewmembers cited differences in the cabin environment compared to earlier experience as well as allergic responses to the cabin environment. One of the noted observations was a high level of visible dust in the ISS Node 3 cabin, to the extent it was sticking to the walls. Flight surgeons indicated that this had been reported not just in Node 3 but also throughout the US on-orbit segment and expressed a concern for crew health. Dust on the ISS is expected, with humans being major contributors (via skin shedding, eating, exercising, etc.). Other sources such as on-orbit maintenance activities can release dust from sources such as payloads and systems, clothing, and visiting vehicles. As a precautionary measure, in the middle of 2012, an investigation was launched to define and mitigate dust sources and to determine if exposure to dust might elicit an adverse effect on crew health. As a result of these crewmember reports, particulate and fiber debris samples were collected during ISS Expedition 31 using a handheld portable vacuum cleaner and returned to Earth aboard Soyuz flight 29S in early July 2012 for analyses. Details of the novel “Prime” vacuum cleaner developed for NASA habitats were reported elsewhere [55]. Portions of the samples were aseptically collected for microbiological testing at NASA’s Johnson Space Center (JSC), with the remaining bag and its contents repacked in a biological hood, sealed, and shipped to NASA’s Marshall Space Flight Center (MSFC) for particle size testing, as well as to JPL to determine their molecular microbiological composition. The vacuum cleaner bag was shipped via FedEx at room temperature with instructions that the samples not be irradiated in transport.

### Earth analogs

For comparison, particles collected from two JPL cleanroom floors using a Nilfisk GM80CR vacuum cleaner (the disposable bag part number is 81620000; Morgantown, PA) were analyzed (Table 1). Cleanroom certification is based on the maximum number of particles greater than 0.5 μm per cubic foot of air. The air within Class 1 K cleanrooms is maintained at fewer than 1000 particulates per cubic foot, Class 10 K cleanrooms are allowed to harbor a density of 10,000 particles per cubic foot, and so on. The cleanroom samples were from (a) the JPL-SAF cleanroom floor where various Mars mission spacecraft were built (Class 10 K; JPL-SAF Debris) and (b) JPL building 103 where non-mission critical activities were conducted (Class 1 K; JPL-103 Debris). The vacuum cleaner bag debris from the JPL-SAF and JPL-103 buildings was transported to the microbiology laboratories for storage. Sample processing was carried out immediately after aseptic collection of the materials, usually within 7 days from the retrieval of the vacuum bags from the cleanrooms. Both Earth-analog samples were processed at the same time as the ISS Debris samples. However, the ISS HEPA sample was received ~3 months later and hence was analyzed separately.

### Sample processing

All samples were subjected to a variety of microbiological and molecular techniques to elucidate composition of cultivable, viable, and total microorganisms. Independent of the samples taken to cultivate bacterial and fungal analyses, subsamples of the same samples were taken for DNA extraction. Weighing vacuum debris samples was possible, whereas the HEPA filter elements were divided into small pieces and particulates associated with the pieces were aseptically collected using sterile scalpels before being quantitatively measured. Approximately 1 g of each vacuum debris and HEPA filter associated particle was weighed and placed into a sterile tube containing 25 mL of sterile phosphate-buffered saline (PBS) and vortexed for 1 min. After vigorous mixing, large particles were allowed to settle, and aliquots of samples were carefully siphoned and allocated for culture-based (1 mL) and culture-independent analyses (15 mL).

### Traditional culture-based microbial examination

For estimating bacterial populations, after suitable serial tenfold dilution in sterile PBS, 100 μL of the sample suspension was spread onto two plates of R2A media (BD Difco, Franklin Lakes, NJ) and incubated at 25 °C for 2–7 days. For the fungal population enumeration, 100 μL was spread onto two plates of potato dextrose agar (PDA, BD Difco, Franklin Lakes, NJ) and incubated at 25 °C for 2–7 days. Bacterial and fungal colony-forming units (CFUs) were counted and reported as CFU/g of material. Identifications and phylogenetic affiliations were carried out via sequencing for both bacteria and fungi by targeting the 16S rRNA gene [42] and the ITS region [56], respectively. When identifications were ambiguous, sequencing of an additional gene (*gyrB*) was performed to confirm the phylogenetic identity of the purified bacterial isolates [57, 58]. The nucleotide sequences of bacteria (KT763339–KT763368) and fungi (KT832780-KT832790) were deposited in GenBank.

### Sample processing for molecular analysis

The biological materials associated with each sample (15 mL) were further concentrated using Amicon Ultra-50 Ultracel centrifugal filter tubes (Millipore, Billerica, MA). Each filter unit has a molecular mass cutoff of 50 kDa, which facilitates the concentration of microbial cells, spores, and exogenous nucleic acid fragments greater than 100 bp in a final volume of 2.5 mL. All filtered samples were then divided into three separate aliquots: the first aliquot (1000 μL) was subjected to PMA pretreatment (viability assessment), the second (1000 μL) was an untreated environmental sample (viable + nonviable; total DNA), and the third (500 μL) was used for adenosine triphosphate (ATP) analysis (see below).

For measuring viable microbial population, one aliquot of filter-concentrated sample suspension (1000 μL) was treated with 12.5 μL of PMA (2 mM; Biotium, Inc., Hayward, CA) to a final concentration of 25 μM [26, 32], followed by thorough mixing and incubation in the dark for 5 min at room temperature [15]. The sample was exposed to PhAST blue-Photo activation system for tubes (GenIUL, S.L., Terrassa, Spain) for 15 min (in parallel with the non-PMA-treated sample). This step enabled blocking DNA from dead cells [15]. The samples were then split in half and one half was subjected to bead beating with the Fastprep-24 bead-beating instrument (MP Biomedicals, Santa Ana, CA) with parameters set at 5 m/s for 60 s. The second half of the unprocessed sample was then combined with the mechanically disrupted counterpart before DNA was extracted via the Maxwell 16 automated system (Promega, Madison, WI), in accordance with manufacture instructions [59]. Resulting DNA suspensions (100 μL each) were stored at −20 °C.

### Quantitation of total and viable microorganisms using molecular methods

#### ATP assay

A bioluminescence assay was performed to determine the total ATP and intracellular ATP from all samples using the CheckLite HS kit (Kikkoman Corp., Noda, Japan), as described previously [60]. Briefly, to determine the total ATP (dead and viable microbes), 100-μL sample aliquots (four replicates) were each combined with 100 μL of a cell lysing detergent (benzalkonium chloride) then incubated at room temperature for 1 min prior to the addition of 100 μL of a luciferin-luciferase reagent. The sample was mixed, and the resulting bioluminescence was measured with a luminometer, the Lumitester K-210 (Kikkoman Corp.). To determine intracellular ATP (viable microbes), 50 μL of an ATP-eliminating reagent (apyrase, adenosine deaminase) was added to a 500-μL portion of the sample and allowed to incubate for 30 min to remove any extracellular ATP, after which the assay for ATP was carried out, as described above, in four replicates, including sterile PBS as negative controls. As previously established, one RLU, the unit of measurement of ATP, was assumed to be approximately equal to one CFU [42].

#### qPCR assay

Real-time quantitative polymerase chain reaction (qPCR) assay, targeting the 16S rRNA gene, was performed in triplicate with a CFX-96 thermal cycling Instrument (Bio-Rad, Hercules, CA) to quantify the bacterial burden. Bacteria-directed primers targeting the 16S rRNA gene 1369 F (5′-CGG TGA ATACGT TCY CGG-3′) and modified 1492R (5′-GGW TAC CTTGTT ACG ACT T-3′) were used for this analysis [61]. Each 25-μL reaction consisted of 12.5 μL of 2X iQ SYBR Green Supermix (BioRad, Hercules, CA), 1 μL each of forward and reverse oligonucleotide primers (10 μM each), and 1 μL of template DNA. Purified DNA from *B. pumilus* cells served as the positive control and DNase/RNase-free molecular-grade distilled water (UltraPure, Gibco) was used as the negative control. These controls were included in all qPCR runs. Reaction conditions were as follows: a 3-min denaturation at 95 °C, followed by 35 cycles of denaturation at 95 °C for 15 s, and a combined annealing and extension at 55 °C for 35 s.

### Molecular microbial diversity analysis using next-generation sequencing methods

#### Pyrosequencing conditions

Bacterial primers 28 F (5′-GAG TTT GAT CNT GGC TCA G-3′) and 519R (5′-GTN TTA CNG CGG CKG CTG-3′) were used to amplify ~500-bp fragments spanning the V1–V3 hypervariable regions of the bacterial 16S rRNA gene. Archaeal primers 341 F (5′-GYG CAS CAG KCG MGA AW-3′) and 958R (5′-GGA CTA CVS GGG TAT CTA AT-3′) were used to amplify ~600-bp fragments spanning the V3–V5 hypervariable regions of the archaeal 16S rRNA gene. A fungal primer set ITS1F (5′-CTT GGT CAT TTA GAG GAA GTA A-3′) and ITS4R (5′-TCC TCC GCT TAT TGA TAT GC-3′) was employed to amplify ~600-bp fragments of the fungal ITS region. These primer pairs were tailored for pyrosequencing by adding a fusion linker and a proprietary 8-nt barcode sequence at the 5′ end of the forward primer and a biotin and fusion linker sequence at the 5′ end of the reverse primer [62]. A HotStarTaq Plus master mix kit (Qiagen, Valencia, CA) was used to catalyze the PCR under the following thermal cycling conditions: initial denaturing at 95 °C for 5 min, followed by 35 cycles of denaturing at 95 °C for 30 s, annealing at 54 °C for 40 s, and extension at 72 °C for 1 min, finalized by a 10-min elongation at 72 °C. Resulting PCR products were purified via Diffinity Rapid Tip (Diffinity Genomics, Inc., West Henrietta, NY) chemistry and were then pooled accordingly. Small fragments were removed with Agencourt Ampure Beads (Beckman Coulter, Brea, CA).

In preparation for FLX-Titanium sequencing (Roche, Nutley, NJ), resulting PCR amplicon fragment size and concentration were accurately measured with DNA 1000 chips using a Bioanalyzer 2100 automated electrophoresis station (Agilent, Santa Clara, CA) and a TBS-380 Fluorometer (Turner Biosystems, Sunnyvale, CA). The total volume of initial PCR product used for subsequent emulsion PCR was 2 μL for strong positives (>10 ng/μL), 5 μL for weak positives (5 to 10 ng/μL), and 20 μL for samples that failed to yield PCR products (<5 ng/μL). This normalization step helped to ensure minimal bias favoring downstream amplification from initially strong PCR products. Approximately 9.6 × 10^6^ molecules of ~600-bp double-stranded DNA were combined with 9.6 × 10^6^ DNA capture beads and then subjected to emulsion PCR conditions. Following recovery and enrichment, bead-attached DNA molecules were denatured with NaOH and sequencing primers were annealed. A four-region 454 pyrosequencing run was performed on a GS PicoTiterPlate (PTP) using the Genome Sequencer FLX System, in accordance with manufacturer instructions (Roche, Nutley, NJ). Twenty-four to 30 tagged samples were applied to each quarter region of the PTP. All pyrosequencing procedures were performed at the Research and Testing Laboratory (Lubbock, TX) in accordance with well-established protocols [62].

#### Illumina sequencing conditions

The library preparations for next-generation sequencing and Illumina MiSeq sequencing were conducted by GENEWIZ, Inc. (South Plainfield, NJ). The DNA samples were quantified using a Qubit 2.0 fluorometer (Invitrogen, Carlsbad, CA) and DNA quality was confirmed by electrophoresis (0.8 % agarose gel). The sequencing library was constructed using a MetaVx™ Library Preparation kit (GENEWIZ, Inc., South Plainfield, NJ). In brief, 5–50 ng of DNA was used for amplicon generation to cover the V3 and V4 hypervariable regions of 16S rDNA. Indexed and universal adapters were added to the ends of the 16S rDNA amplicons by limited-cycle PCR. The DNA libraries were validated with an Agilent 2100 Bioanalyzer (Agilent Technologies, Palo Alto, CA) and quantified using Qubit and real-time PCR (Applied Biosystems, Carlsbad, CA). The DNA libraries were multiplexed and loaded on an Illumina MiSeq following the instructions from the manufacturer (Illumina, San Diego, CA). A 2 × 150 paired-end (PE) configuration was used for sequencing. The image analysis and base calling were processed using MiSeq Control Software. The initial taxonomy was performed on Illumina’s BaseSpace cloud computing platform.

#### Bioinformatic analyses of bacterial pyrosequences

High-throughput 16S rRNA sequencing data were processed. Bacterial and archaeal sequences were processed and analyzed using the mothur software [16, 63]. Sequences were quality filtered by removing sequences that (a) did not contain the primer sequence, (b) contained an uncorrectable barcode, (c) were <250 nt in length, (d) had homopolymers longer than 8 nt, or (e) had a quality score of <25; and then demultiplexed using the respective sample nucleotide barcodes. These sequences were searched against the Greengenes reference database [64, 65] and clustered into OTUs based on their sequence similarity (97 %) with UCLUST [66]. A representative sequence was picked from each OTU and taxonomic classification was assigned using mothur’s Bayesian classifier [67] and Greengenes training sequences and taxonomy [64, 65].

#### Bioinformatic analyses of fungal pyrosequences

The sequences were run through ITSx 1.0.9 [68] to remove non-ITS sequences, assembly chimeras, and sequences for which none of the 3′ end of SSU rDNA or the 5′ end of 5.8S rDNA was detected. The ITS1 sub-region was extracted from the remaining sequences for further analyses. Chimera control was exercised through UCHIME 7 [69] using the UNITE chimera reference dataset [70] as reference corpus. The sequences were subjected to clustering and taxonomic assignment in the Sequence Clustering and Analysis of Tagged Amplicons (SCATA) next-generation sequencing analysis pipeline (http://scata.mykopat.slu.se) using the February 2014 release of UNITE as taxonomic reference corpus. The default sequence quality control settings of SCATA were used; however, the clustering threshold was set to 98.5 % [71]. All taxonomic affiliations proposed by SCATA were examined manually using Basic Local Alignment Search Tool (BLAST) 2.2.30 [72] in GenBank [73] and UNITE [74] and occasionally refined using the principles outlined in Koljalg et al. [75] and Nilsson et al. [76].

#### Preprocessing of bacterial Illumina sequences

The 12 sets of 16S rDNA V3 amplicon data were sequenced at GENEWIZ, Inc. (South Plainfield, NJ,), where the obtained reads were trimmed to remove primer sequences. The raw sequence dataset was composed of 10,241,173 paired-end reads, 150 bp in length, with exceptionally high quality (<0.1 % error rate) (Resphera Discovery^TM^ protocol, Baltimore, MD). After trimming noisy reads and removal of low-quality and chimeric sequences, we identified a moderate level of expected contaminants, including chloroplast and mammalian mitochondrial sequence (range 0.0001–9.3 %), as well as a low level of unknown contaminants (range 0.01–1.8 %). Exploratory characterization of unknown contaminant representatives indicated nonspecific amplification mitochondrial sequences from various eukaryotic organisms. After completion of preprocessing, the resulting high-quality R1 and R2 read sets contained 9.1 M and 9.3 M sequences, respectively, with an average length of 128 bp. Due to the primer set and sequencing technology selected by the vendor, we were unable to merge paired-end sequences into longer consensus fragments as they did not overlap. Therefore, in this analysis, we compared our findings between the R1 (forward)- and R2 (reverse)-associated datasets to evaluate consistency. Across R1 and R2 datasets, each sample had on average 917,060 clean sequences. After clustering sequences into OTUs, OTU tables were rarefied to an even coverage of 525,000 sequences per sample. On average, Good’s coverage statistic for rarefied samples was 99.78 %, indicating we have observed the vast majority of OTU diversity in each community.

#### Bioinformatic analysis of bacterial Illumina sequences

Sequences were de-multiplexed using 5′ barcode identifiers and analyzed using the Resphera Discovery™ protocol (Baltimore, MD). Briefly, 16S rRNA sequence fragments were first screened in Quantitative Insights into Microbial Ecology (QIIME) [77] for quality and length requiring: (a) trimming at the first 5-bp run of Phred quality scores below 20, (b) one ambiguous base call or less, and (c) a minimum final length of 125 bp after trimming of forward and reverse primer sequences. Passing sequences were screened for PhiX-174 spiked fragments and PCR-associated chimeras by UCHIME [69] (de novo mode). Non-chimeric reads were then filtered for contaminant chloroplast and mitochondrial sequences using the RDP classifier [17], followed by a broad nucleotide BLAST (BLASTN) [78] search of the GreenGenes 16S rRNA reference database [65] (v1.1) to identify potential unknown contaminants. The resulting high-quality dataset was clustered into de novo OTUs using UCLUST [66] with a 95 % identity threshold. OTU representatives were assigned to a taxonomic lineage using the RDP classifier trained on the Resphera 16S rRNA database (v1.1) requiring a minimum confidence score of 0.50.

#### Statistical analyses

To perform pairwise statistical comparisons of sample groups of interest, a negative binomial test implemented in DESeq [79] or Fisher’s exact test was utilized and controlled for false positives using the false discovery rate (FDR) [80]. For the comparison of bacterial pyrosequence and Illumina data and fungal pyrosequence analysis, multivariate statistical analyses of community profiles were performed using an “in-house R-script” employing the libraries vegan, ape, gplots, mgcv, and GUniFrac [81]. First, each dataset composed of OTU abundances per sample was rarefied to the lowest number of reads and a Bray-Curtis index was calculated. This procedure was repeated 10,000 times and the average Bray-Curtis distance was calculated for each dataset in order to avoid biases arising from rarefication. The Bray-Curtis distance was then utilized to calculate principal coordinate (PCoA) analyses, PERMANOVA (1000 permutations), and MRPP (1000 permutations).

## Availability of supporting data

The data set supporting the results of this article is available in the NCBI SRA repository, under SRA #280254.

## Additional files

Additional file 1: Table S1. Cultivable bacterial species isolated from the ISS vacuum cleaner bag and associated bag. **Table S2.** Pyrosequencing-based and Illumina-based phyla present in ISS and Earth cleanroom samples. **Table S3.** Identification of viable sequences in ISS samples for the cultivable isolates. **Table S4.** Significance overview. PERMANOVA and MRPP provide significant *P* values for ISS and Earth cleanroom microbiome irrespective of the dataset used. Viability assay did not show a significant difference in the observed community relationships. Adonis = PERMANOVA *P* value, MRPP - A = chance corrected within-group agreement. (DOC 189 kb) Additional file 2: Figure S1. Pyrosequencing-based (*left*) and Illumina-based (*right*) phyla present in ISS and Earth cleanroom samples. (PNG 550 kb) Additional file 3: Figure S2. Independent analyses of bacterial sequences derived from pyrosequencing and Illumina sequencing revealed a significant difference in the community profile of ISS and Earth cleanroom microbiome. These differences are displayed in ordination analyses. (JPEG 146 kb)
