# Supplementary material for: Microbiomes of the dust particles collected from the International Space Station and Spacecraft Assembly Facilities
Source: Microbiome. 2015 Oct 27;3:50. doi: 10.1186/s40168-015-0116-3 (PMC4624184; doi:10.1186/s40168-015-0116-3)
Supplement: Additional file 1: Table S1. — Cultivable bacterial species isolated from the ISS vacuum cleaner bag and associated bag. Table S2. Pyrosequencing-based and Illumina-based phyla present in ISS and Earth cleanroom samples. Table S3. Identification of viable sequences in ISS samples for the cultivable isolates. Table S4. Significance overview. PERMANOVA and MRPP provide significant P values for ISS and Earth cleanroom microbiome irrespective of the dataset used. Viability assay did not show a significant difference in the observed community relationships. Adonis = PERMANOVA P value, MRPP - A = chance corrected within-group agreement. (DOC 189 kb) [file 40168_2015_116_MOESM1_ESM.doc]

Additional file 1

Table S1. Cultivable bacterial species isolated from the ISS vacuum cleaner bag and associated bag.

| Species | | ISS HEPA Total | ISS Debris Total | JPL-SAF Debris Total | JPL-103 Debris Total |
| --- | --- | --- | --- | --- | --- |
| Bacteria (41 strains, 29 recognized species) | |  |  |  |  |
|  | *Agrococcus jenensis* | × |  |  |  |
|  | *Agrococcus lahaulensis* | × |  |  |  |
|  | *Arthrobacter agilis* | × |  |  |  |
|  | *Bacillus altitudinis* |  |  | × |  |
|  | *Bacillus anthracis-cereus* group*** | 5 |  |  |  |
|  | *Bacillus aquimaris* | × |  |  |  |
|  | *Bacillus arbutinivorans* | × |  |  |  |
|  | *Bacillus firmus* | × |  |  |  |
|  | *Bacillus fusiformis* |  | × |  |  |
|  | *Bacillus ginsengihumi* |  | × | × |  |
|  | *Bacillus licheniformis* |  |  |  | × |
|  | *Bacillus megaterium* |  | **×** |  |  |
|  | *Bacillus simplex* |  |  | × |  |
|  | *Bacillus* sp. |  | × |  |  |
|  | *Brevibacillus borstelensis* |  |  |  | × |
|  | *Brevibacillus* sp. |  | × |  |  |
|  | *Brevundimonas vesicularis* | × |  |  |  |
|  | *Dietzia lutea* | × |  |  |  |
|  | *Kocuria rosea* | × |  | × |  |
|  | *Massilia brevitalea* |  |  |  | × |
|  | *Masillia* sp. | × |  |  |  |
|  | *Microbacterium foliorum* | × |  |  |  |
|  | *Paenibacillus* sp. |  | × |  |  |
|  | *Paenibacillus taichungensis* | × |  |  |  |
|  | *Pantoea agglomerans* |  |  | × |  |
|  | *Paracoccus sp.* |  |  |  | × |
|  | *Pontibacter populi* | × |  |  |  |
|  | *Pseudomonas luteola* |  | × |  |  |
|  | *Pseudomonas rhizosphaerae* |  |  | × |  |
|  | *Solibacillus silvestris* | 2 |  |  |  |
|  | *Staphylococcus cohnii* |  | × |  |  |
|  | *Staphylococcus epidermidis* |  | × |  |  |
|  | *Staphylococcus hominis* |  | × |  |  |
|  | *Staphylococcus warneri* |  | × |  |  |
| Fungi | (19 strains, 6 recognized species) |  |  |  |  |
|  | *Aspergillus candidus* |  |  | × |  |
|  | *Aspergillus niger* | 6 | × |  |  |
|  | *Aspergillus* sp. |  | × |  |  |
|  | *Aspergillus terreus* | × |  | × |  |
|  | *Aspergillus unguis* |  | × |  |  |
|  | *Glomus sp.* | × |  |  |  |
|  | *Mucor* sp. |  | × |  |  |
|  | *Penicillium camemberti* | × |  |  |  |
|  | *Penicillium janthinellum* |  |  |  | × |
|  | *Penicillium* sp. |  | × |  |  |
|  | *Rhodotorula* sp. |  | × |  |  |
|  | Unidentified fungi |  | × |  |  |

* Not able to differentiate these species. The molecular analyses revealed that *pXO1* and *pXO2* genes were not present (data not shown).

Table S2. Pyrosequencing-based and Illumina-based phyla present in ISS and Earth cleanroom samples.

| Phylum | Number of bacterial pyrosequences retrived from: | | | | | | | |  | Number of bacterial sequences retrieved from Illumina sequencing: | | | | | | | |
| --- | --- | --- | --- | --- | --- | --- | --- | --- | --- | --- | --- | --- | --- | --- | --- | --- | --- |
|  | ISS HEPA Total | ISS HEPA Viable | ISS Debris Total | ISS Debris Viable | JPL-SAF Debris Total | JPL-SAF Debris Viable | JPL-103 Debris Total | JPL-103 Debris Viable |  | ISS HEPA Total | ISS HEPA Viable | ISS Debris Total | ISS Debris Viable | JPL-SAF Debris Total | JPL-SAF Debris Viable | JPL-103 Debris Total | JPL-103 Debris Viable |
| Acidobacteria |  |  | 5 |  | 11 | 1 | 5 | 27 |  | 14 | 1 |  | 26 | 3724 | 5488 | 1517 | 1517 |
| Actinobacteria | 1,368 | 646 | 19,571 | 16,320 | 1,615 | 723 | 544 | 11 |  | 350057 | 559824 | 465170 | 742867 | 296193 | 371869 | 116353 | 85326 |
| Armatimonadetes |  |  |  |  | 18 | 3 | 1 |  |  | 14 | 30 |  |  | 434 | 567 | 22 | 2 |
| Bacteroidetes | 34 | 1 | 136 | 1 | 314 | 98 | 136 | 7 |  | 40933 | 1477 | 31930 | 18064 | 88795 | 63764 | 24616 | 9942 |
| Chloroflexi |  |  |  |  | 22 | 12 | 4 |  |  | 30 | 65 | 457 | 543 | 7183 | 9815 | 741 | 536 |
| Cyanobacteria | 6 |  | 270 | 51 | 1,157 | 1,409 | 50 |  |  | 5223 | 325 | 34677 | 142 | 70937 | 141950 | 5278 | 867 |
| Deinococcus-Thermus |  |  |  |  | 52 | 20 | 21 |  |  | 62 | 44 | 611 |  | 16589 | 21683 | 11244 | 10862 |
| Firmicutes | 269 | 1 | 9,690 | 1,439 | 299 | 324 | 111 |  |  | 137334 | 23323 | 524310 | 317940 | 79275 | 162687 | 32285 | 3896 |
| Fusobacteria | 3 |  | 130 |  | 2 |  | 1 |  |  | 1859 |  | 10754 |  | 360 | 209 | 103 |  |
| Gemmatimonadetes |  |  |  |  | 21 | 9 | 27 | 4 |  |  |  | 444 |  | 1172 | 1257 | 1003 | 1150 |
| Planctomycetes |  |  |  |  |  |  | 5 |  |  | 1 |  | 24 |  | 4 | 2 | 23 | 53 |
| Proteobacteria | 28 | 7 | 265 | 108 | 4,898 | 3,898 | 4,144 | 117 |  | 15550 | 2391 | 70698 | 36162 | 451082 | 670793 | 281521 | 270456 |
| Spirochaetes |  |  |  |  |  |  |  |  |  | 8 |  | 78 |  |  |  | 1 |  |
| SR1 |  |  | 4 |  |  |  |  |  |  | 15 |  | 82 |  | 3 |  |  |  |
| Tenericutes |  |  |  |  |  | 3 |  |  |  |  |  |  |  | 64 | 6 |  |  |
| TM7 |  |  | 27 |  | 7 | 2 |  |  |  | 360 | 23 | 5727 |  | 1166 | 1503 | 190 | 10 |
| Verrucomicrobia |  |  |  |  | 4 | 1 |  |  |  | 22 |  | 65 | 1 | 45 | 219 | 18 | 1 |
|  |  |  |  |  |  |  |  |  |  |  |  |  |  |  |  |  |  |

Table S3. Identification of viable sequences in ISS samples for the cultivable isolates.

|  |  | Cultivable species that were isolated from: | | Viable pyrosequences retrieved from: | | Viable sequences retrieved from Illumina: | |
| --- | --- | --- | --- | --- | --- | --- | --- |
| Cultivable species during this study | | HEPA | Debris | HEPA | Debris | HEPA | Debris |
| Bacteria |  |  |  |  |  |  |  |
|  | *Agrococcus jenensis* | × |  |  |  | × |  |
|  | *Agrococcus lahaulensis* | × |  |  |  | × |  |
|  | *Arthrobacter agilis* | × |  |  |  | × | × |
|  | *Bacillus anthracis – cereus* group | 5 |  |  |  | × | × |
|  | *Bacillus aquimaris* | × |  |  |  | × | × |
|  | *Bacillus arbutinivorans* | × |  |  |  | × | × |
|  | *Bacillus firmus* | × |  |  |  | × | × |
|  | *Bacillus fusiformis* |  | × |  |  | × | × |
|  | *Bacillus megaterium* |  | × |  |  | × | × |
|  | *Bacillus* sp. |  | × |  |  | × | × |
|  | *Brevibacillus* sp. |  | × |  |  |  |  |
|  | *Brevundimonas vesicularis* | × |  |  |  | × | × |
|  | *Dietzia lutea* | × |  |  |  | × | × |
|  | *Kocuria rosea* | × |  |  |  | × | × |
|  | *Masillia* sp. | × |  |  |  |  |  |
|  | *Microbacterium foliorum* | × |  |  |  | × | × |
|  | *Paenibacillus* sp. |  | × |  |  | × | × |
|  | *Paenibacillus taichungensis* | × |  |  |  | × | × |
|  | *Pontibacter populi* | × |  |  |  |  |  |
|  | *Pseudomonas luteola* |  | × |  | × | × | × |
|  | *Solibacillus silvestris* | 2 |  |  |  |  |  |
|  | *Staphylococcus cohnii* |  | × |  | × | × | × |
|  | *Staphylococcus epidermidis* |  | × |  | × | × | × |
|  | *Staphylococcus hominis* |  | × |  | × | × | × |
|  | *Staphylococcus warneri* |  | × |  | × | × | × |
|  | Unidentified bacteria | 2 |  |  | × |  |  |
|  |  |  |  |  |  |  |  |
| Fungi | *Aspergillus niger* | 6 | × |  |  |  |  |
|  | *Aspergillus* sp. |  | × |  |  |  |  |
|  | *Aspergillus terreus* | × |  |  |  |  |  |
|  | *Aspergillus unguis* |  | × |  |  |  |  |
|  | *Glomus sp.* | × |  |  |  |  |  |
|  | *Mucor* sp. |  | × |  |  |  |  |
|  | *Penicillium camemberti* | × |  |  |  |  |  |
|  | *Penicillium* sp. |  | × |  | × |  |  |
|  | *Rhodotorula* sp. |  | × |  |  |  |  |
|  | Unidentified fungi |  | × |  |  |  |  |

| Table S4. Significance overview. PERMANOVA and MRPP provide significant p-values for ISS and Earth cleanroom microbiome irrespective of the dataset used. Viability assay did not show a significant difference in the observed community relationships. Adonis = PERMANOVA p-value, MRPP - A = chance corrected within-group agreement. | | | | | | |
| --- | --- | --- | --- | --- | --- | --- |
|  | **Location: ISS versus Cleanroom** | | | **Viability: Viable versus Total** | | |
|  | **Adonis** | **MRPP - delta** | **MRPP - A** | **Adonis** | **MRPP - delta** | **MRPP - A** |
| **Bacteria (454)** | 0.020 | 0.024 | 0.517 | 0.965 | 0.969 | -0.100 |
| **Bacteria (MiSeq)** | 0.020 | 0.029 | 0.255 | 0.819 | 0.846 | -0.074 |
| **Fungi** | 0.033 | 0.036 | 0.109 | 0.593 | 0.593 | -0.026 |
