# Supplementary figures and images for: Microbiomes of the dust particles collected from the International Space Station and Spacecraft Assembly Facilities
Source: Microbiome. 2015 Oct 27;3:50. doi: 10.1186/s40168-015-0116-3 (PMC4624184; doi:10.1186/s40168-015-0116-3)

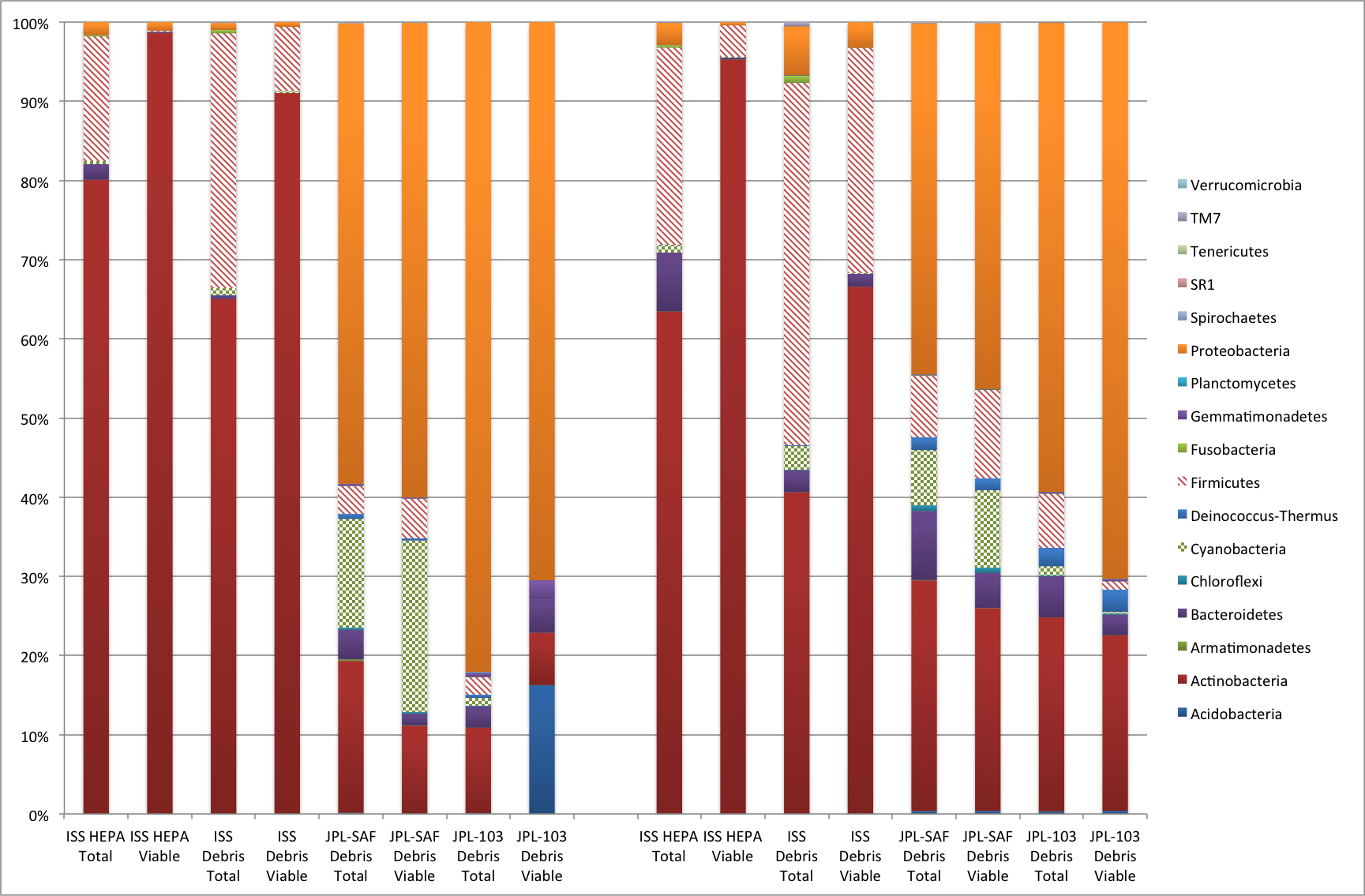

Supplement: Additional file 2: Figure S1. — Pyrosequencing-based (left) and Illumina-based (right) phyla present in ISS and Earth cleanroom samples. (PNG 550 kb) [file 40168_2015_116_MOESM2_ESM.png]

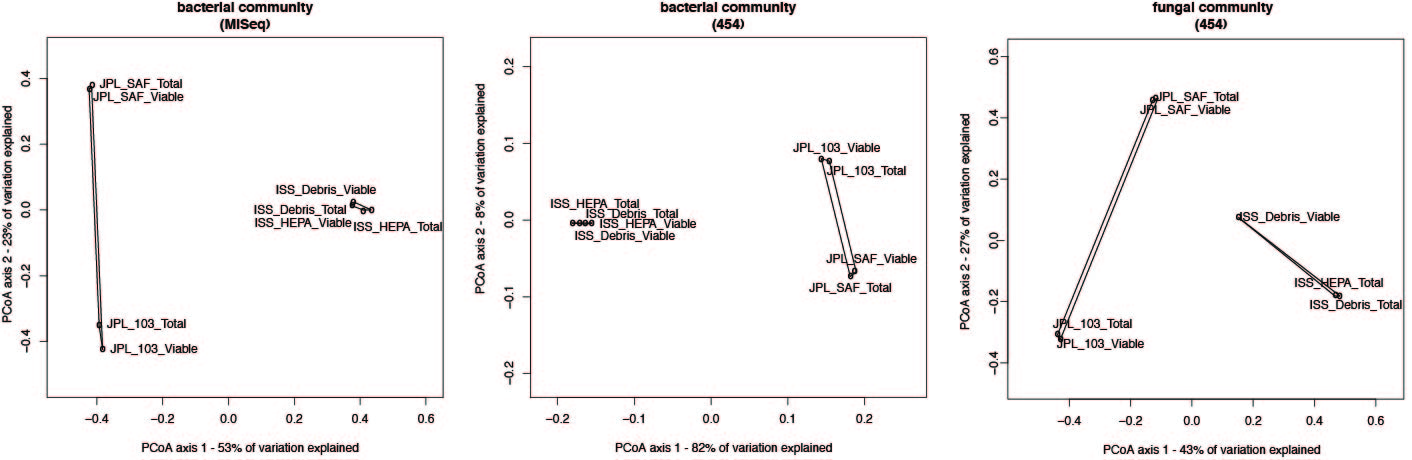

Supplement: Additional file 3: Figure S2. — Independent analyses of bacterial sequences derived from pyrosequencing and Illumina sequencing revealed a significant difference in the community profile of ISS and Earth cleanroom microbiome. These differences are displayed in ordination analyses. (JPEG 146 kb) [file 40168_2015_116_MOESM3_ESM.jpg]
